# Evaluation of semi-quantitative scoring of Gram staining or semi-quantitative culture for the diagnosis of ventilator-associated pneumonia: a retrospective comparison with quantitative culture

**DOI:** 10.1186/2052-0492-1-2

**Published:** 2013-10-23

**Authors:** Soshi Hashimoto, Nobuaki Shime

**Affiliations:** Department of Anesthesiology and Intensive Care, Kyoto Prefectural University of Medicine, Kyoto, 602-8566 Japan; Department of Emergency and Critical Care Medicine, National Hospital Organization Kyoto Medical Center, Kyoto, 612-8555 Japan

**Keywords:** Gram stain, Semi-quantitative culture, Quantitative culture, Ventilator-associated pneumonia

## Abstract

**Background:**

Semi-quantitative Gram stain and culture methods are still commonly used for diagnosing ventilator-associated pneumonia (VAP), due to its convenience. Only a few studies, however, have assessed the reliability of these methods when compared with quantitative cultures, a current standard for the diagnosis of VAP. The objective of this study was to assess the utility of semi-quantitative scores obtained using Gram stains and cultures of endotracheal aspirates when compared with quantitative cultures in the diagnosis of VAP.

**Methods:**

A retrospective chart review of mechanically ventilated patients with clinically suspected VAP in a single intensive care unit was performed. Semi-quantitative scores of Gram stains or culture results were compared with quantitative culture results of endotracheal aspirate for the diagnosis of VAP in 136 samples for 51 patients.

**Results:**

The semi-quantitative scores of Gram stains and the semi-quantitative culture results significantly correlated with the log value of the quantitative culture results (*r*_s_ = 0.64 and 0.75). When using a log count ≥6 of quantitative cultures as the reference standard for the diagnosis of VAP, the sensitivity and specificity was 95% and 61% for Gram stain score of ≥1+, and was 42% and 96% for Gram stain score ≥3+, respectively. The sensitivity and specificity was 96% and 40% for the semi-quantitative culture score of ≥2+, and was 59% and 86% for the semi-quantitative culture score of ≥3+, respectively.

**Conclusions:**

Absence of bacteria in semi-quantitative Gram stain and poor growth (≤1+) in semi-quantitative culture method could be utilized to exclude the possibility of VAP, whereas detection of abundant (≥3+) bacteria in semi-quantitative Gram stain could be utilized to strongly suspect VAP.

## Background

Ventilator-associated pneumonia (VAP) is associated with increased mortality, morbidity, and medical costs [1–3]. Appropriately diagnosing VAP is crucial as it guides appropriate antimicrobial therapy followed by improved outcome. For the microbiological diagnosis, Gram stain and subsequent culture methods for respiratory tract secretions obtained using either endotracheal aspiration or fiberoptic bronchoscopy have widely been used [3–6]. Of those, the use of quantitative culture has been a current standard for the diagnosis of VAP [4]. Problems associated with the quantitative culture method, however, are too cumbersome, labor intensive, and costly [7, 8].

Semi-quantitative Gram stain and culture are subjective quantitative scoring methods of bacterial load in Gram stain sample or culture plate by microbiological technologist. The benefit of the semi-quantitative method consists of simplicity and lower costs. In this study, we assessed whether the semi-quantitative scoring of Gram staining or semi-quantitative culture could be utilized as an alternative of quantitative culture for the diagnosis of VAP.

## Methods

### Patients

This study was conducted in an intensive care unit of the University Hospital of Kyoto Prefectural University of Medicine. This study was approved by the Ethics Committee on Human Research of the Kyoto Prefectural University of Medicine, and informed consent was waived. Patients clinically suspected for VAP and performed culture examination of respiratory sample were enrolled between November 2007 and June 2011. Patients who suspected with viral pneumonia were not included. The clinical suspicion of VAP was made based on the following CDC criteria [9]: at least one of the signs of (a) elevated body temperature >38°C, (b) blood leukocyte count ≥12,000 or <4,000 per cubic meter, (c) altered metal status without other recognized cause, plus at least two of the signs of (a) new onset of purulent tracheal secretions, (b) increased spontaneous respiratory rate, (c) newly appeared rales or bronchial sounds, or (d) decrease in the PaO_2_/fraction of inspired oxygen (F_I_O_2_) ratio ≤240 or increased oxygen or ventilation demand, plus radiologically confirmed new or persistent pulmonary infiltrate, consolidation or cavitation. The diagnosis of VAP was finally made by microbiological examination using quantitative culture. For the microbiological criteria, a threshold of 10^6^ CFU/ml was used for positive quantitative culture for endotracheal aspirate [4, 10].

### Sampling procedures

Respiratory tract secretions were obtained by either bronchoscope-directed techniques by ICU physicians or blind techniques by nurses. The specimens were immediately transported to the microbiology laboratory. Gram staining was performed on each specimen by experienced microbiological technologists. The semi-quantitative scoring of Gram stain was based on the number of bacteria per high-power (×1,000) oil immersion field: 0 = no bacteria per field; 1+ = less than one bacterium per field; 2+ = 1–5 bacteria per field; 3+ = 6–30 bacteria per field; and 4+ = more than 30 bacteria per field [11].

### Cultures

Samples were processed according to standard culture procedures, and the results were read after 48 h. The semi-quantitative scoring was determined by the four-quadrant method and classified as follows: 0 = no growth; 1+ = rare growth; 2+ = light growth; 3+ = moderate growth; 4+ = heavy growth [12]. Quantitative culture was performed by serial dilution of the respiratory samples. The colony counts were calculated by the number of colonies visible on the agar plate in relation to the dilution and inoculation factors, and results were reported as colony-forming units per milliliter (CFU/mL).

### Analysis

Statistical analysis was performed using a statistical software program (GraphPad Prism version 5.04, GraphPad Software, San Diego, CA, USA). Variables were expressed as median and interquartile range (IQR). Spearman’s rank correlation coefficient was used to assess the association between semi-quantitative Gram stain scores and log values of quantitative cultures, or semi-quantitative scores and log values of quantitative cultures. A *p* value <0.05 was considered to indicate statistical significance. Sensitivity and specificity of semi-quantitative Gram stain scoring or the semi-quantitative scoring was calculated on the basis of different cutoff points for the diagnosis of VAP based on quantitative culture results. Consistencies of diagnosis between the scoring systems and the quantitative culture were also assessed by kappa statistics.

## Results

Demographic and clinical characteristics of subjects are summarized in Table 1. A total of 136 specimens were obtained from 51 patients with clinical diagnosis of VAP. In 79 specimens, the criteria for VAP according to the quantitative culture results were fulfilled. *Pseudomonas aeruginosa* (*n* = 22, 27.8%), methicillin-sensitive *Staphylococcus aureus* (MSSA) (*n* = 12, 15.2%), methicillin-resistant *Staphylococcus aureus* (MRSA) (*n* = 6, 7.6%), and *Enterobacter cloacae* (*n* = 4, 5.1%) were frequently recovered organisms.

**Table 1:** **Clinical characteristics of study population**

Number of patients	51
Male (*n*, %)	30 (59%)
Age (median, IQR)	51 (0–71)
Weight (median, IQR)	42 (4.9–60)
Surgical patients (*n*, %)	41 (80%)
Duration of mechanical ventilation (median, IQR d)	31 (13–104)
ICU stay (median, IQR d)	43 (15–104)
ICU mortality (*n*, %)	19 (37%)
Total hospital mortality (*n*, %)	24 (47%)
Number of samples	136
Antibiotics within 24 h of endotracheal aspiration (*n*, %)	64 (47%)
Use of saline during endotracheal aspiration (*n*, %)	64 (47%)

### Gram stain scores and quantitative culture results

**Figure 1:**
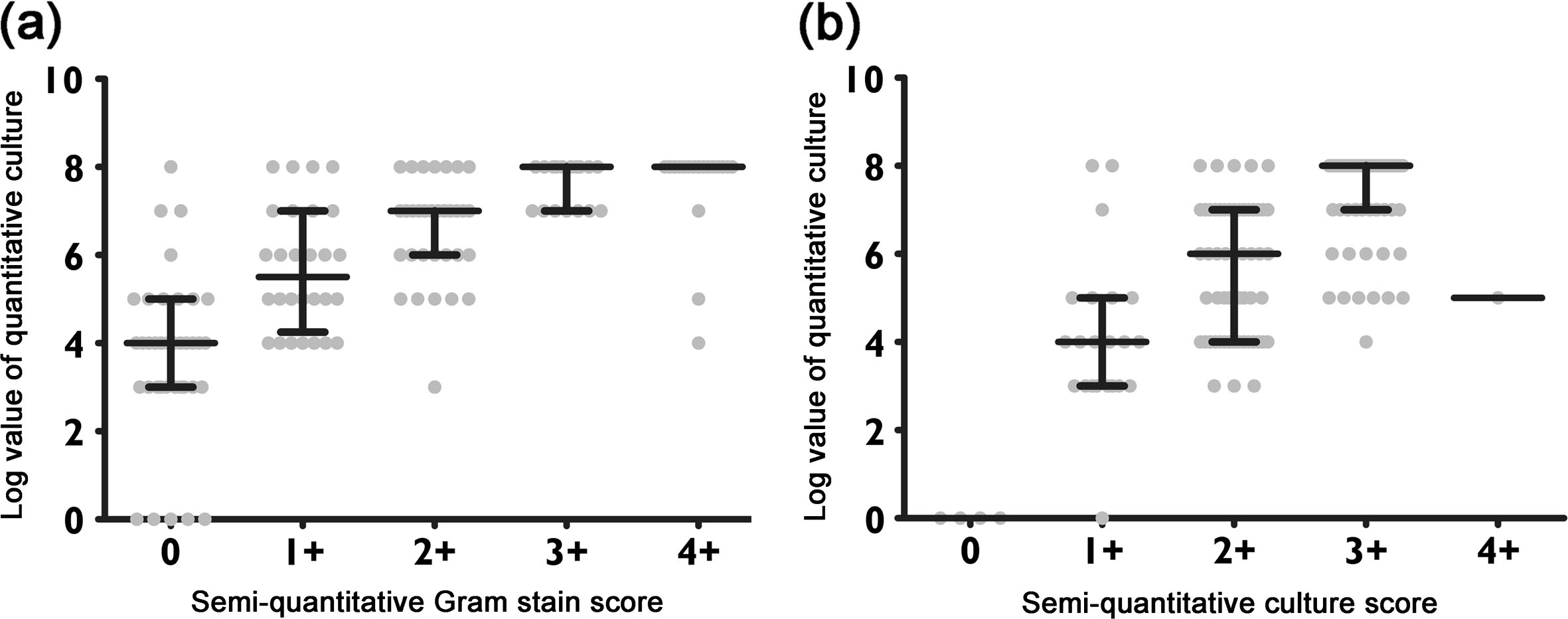
**Comparison between the semi-quantitative Gram stain or culture scoring and quantitative results. (a)** Scatter plot comparing the log count of quantitative culture results and the semi-quantitative Gram stain score; **(b)** scatter plot comparing the log count of quantitative culture results and the semi-quantitative culture score. Each center line represents the median, whereas top and bottom lines denote the interquartile range (IQR, 25th and 75th percentiles).

Of the 136 specimens, semi-quantitative scores of Gram stains were as follows: 39 showed no bacteria, 28 were grade 1+, 34 were grade 2+, 17 were grade 3+, and 18 were grade 4+. Of the 39 specimens that showed no bacteria, 35 (90.0%) had a bacterial count below the VAP diagnostic threshold of 10^6^ CFU/mL. Of the 35 specimens that were grade 3+ or 4+, 33 (94.3%) had a bacterial count above the VAP diagnostic threshold of 10^6^ CFU/mL. The relationship of the semi-quantitative Gram stain score and the quantitative culture of endotracheal aspirate is presented in Figure 1a. A significant correlation was found between the semi-quantitative Gram stain scoring and the quantitative culture technique (*r*_s_ = 0.64, *p* < 0.0001).

### Semi-quantitative and quantitative culture results

Of the 136 specimens, the semi-quantitative culture results were as follows: four showed no growth, 22 were grade 1+, 55 were grade 2+, 54 were grade 3+, and only one was grade 4+. Of these that 26 specimens that showed no growth or were grade 1+, 23 (88.5%) had a bacterial count below the VAP diagnostic threshold of 10^6^ CFU/mL. Of the 55 specimens that were grade 3+ or 4+, 47 (85.5%) had a bacterial count above the VAP diagnostic threshold of 10^6^ CFU/mL. The relationship of the semi-quantitative and the quantitative culture of endotracheal aspirate is presented in Figure 1b. A significant relationship was seen between these two culture techniques (*r*_s_ = 0.75, *p* < 0.0001).

### Sensitivity and specificity, and concordance with quantitative references

The diagnostic test performance of Gram stain is shown in Table 2. When using a log count ≥6 of quantitative culture as a reference standard for the diagnosis of VAP, the sensitivity and specificity of Gram stain score ≥1+ was 95% and 61%, respectively, with a positive predictive value of 77% and a negative predictive value of 90%. Using a Gram stain score ≥3+ as a cutoff point, the sensitivity and specificity was 42% and 96%, respectively, with a positive predictive value of 94% and a negative predictive value of 57%. When we set the cutoff of semi-quantitative Gram stain score at ≥2+, highest agreement with quantitative culture results was observed (kappa = 0.62).

**Table 2:** **Gram stain scoring for the diagnosis of VAP**

	Sensitivity (%)	Specificity (%)	PPV (%)	NPV (%)	PLR	NLR	Kappa (95% CI)
≥1+	95	61	77	90	2.4	0.08	0.59 (0.45–0.72)
≥2+	77	86	88	73	5.5	0.27	0.62 (0.49–0.75)
≥3+	42	96	94	57	10.5	0.6	0.35 (0.23–0.47)
≥4+	20	96	89	47	5	0.83	0.15 (0.05–0.24)

*PPV* positive predictive value, *NPV* negative predictive value, *PLR* positive likelihood ratio, *NLR* negative likelihood ratio.

The diagnostic test performance of semi-quantitative culture is shown in Table 3. When using a log count ≥6 of quantitative culture as a reference standard, the sensitivity and specificity of semi-quantitative score of ≥2+ was 96% and 40%, respectively, with a positive predictive value of 69% and a negative predictive value of 88%. Using a semi-quantitative culture score of ≥3+ as cutoff point, the sensitivity and specificity was 59% and 86%, respectively, with a positive predictive value of 85% and a negative predictive value of 60%. The degree of concordance with quantitative diagnosis was highest (kappa = 0.43) when a semi-quantitative culture score of ≥3+ as cutoff point.

**Table 3:** **Semi-quantitative culture scoring for the diagnosis of VAP**

	Sensitivity (%)	Specificity (%)	PPV (%)	NPV (%)	PLR	NLR	Kappa (95% CI)
≥2+	96	40	69	88	1.6	0.1	0.4 (0.25–0.54)
≥3+	59	86	85	60	4.2	0.48	0.43 (0.29–0.57)

*PPV* positive predictive value, *NPV* negative predictive value, *PLR* positive likelihood ratio, *NLR* negative likelihood ratio.

## Discussion

In this study, a significant correlation was found between the semi-quantitative Gram stain or culture results and quantitative culture results. Moreover, the semi-quantitative Gram stain score of 0 or the semi-quantitative culture score of ≤1+ indicates a low probability of VAP, and the semi-quantitative Gram stain score of ≥ 3+ or the semi-quantitative culture score ≥ 3+ indicates a high probability of VAP, defined as quantitative cultures with ≥10^6^ CFU/mL.

Although the diagnostic value of Gram stain in clinical practice remains controversial [13–16], a recent meta-analysis reported that the negative predictive value of Gram stain is 91%, suggesting that VAP is unlikely if the Gram stain result is negative [17]. Our findings are in line with the results, highlighting the potential merit of withholding antibiotic therapy for patients with clinically suspected VAP if no organism detected on initial Gram stain results. In contrast, a 3+ or more of Gram stain score indicates the necessity of administering empiric antibiotics as it strongly suspect the probability of VAP. The Gram stain scoring guidance might help decision of empiric antibiotic administration and avoid unnecessary antibiotic use [18].

This study also demonstrated a significant correlation between semi-quantitative and quantitative culture results. In a recent single center trial, Riaz et al. [19] compared quantitative and semi-quantitative cultures. With a reference standard of quantitative culture method, the sensitivity of semi-quantitative threshold of ≥1+ (sparse growth) or ≥2+ (moderate growth) was 97% and 85%, respectively, which is comparable to the current study. No or sparse growth on semi-quantitative culture could be used to rule out VAP. On the other hand, heavy growth on semi-quantitative culture (3+) has a high positive predictive value of 85%. Those results indicate the acceptable efficacy of utilizing semi-quantitative threshold for the diagnosis of VAP specifically in resource- or cost-limited situations.

However, differentiating colonizing organisms from infectious organisms is difficult in the case of semi-quantitative score of 2+. Prior studies have also reported that the results of semi-quantitative cultures were not perfectly concordant with cultures that were obtained via invasive quantitative culture methods [12, 20, 21]. It should be noted that the semi-quantitative scoring system cannot completely replace quantitative culture methods for the diagnosis of VAP.

This study has several limitations. First, as this study was performed in a single center, the results might not be extrapolated to other groups with different case-mix or clinical laboratory system. Of note, there are no standardized criteria for Gram stain and semi-quantitative culture interpretation, leading to inter-laboratory variability in the semi-quantitative grading. Second, we did not assess the impact of antibiotic therapy on study results. Antibiotic therapy may affect bacterial growth in culture samples and contribute to false-negative cultures despite the detection on Gram staining. Fagon et al. reported decreased sensitivity and specificity of respiratory cultures in groups of patients that had received antibiotic therapy before sampling [22].

## Conclusions

In summary, semi-quantitative Gram stain or culture scoring methods may partly be used as an alternative of quantitative cultures, a current standard for the diagnosis of VAP. Absence of bacteria in semi-quantitative Gram stain and poor growth (≤1+) in semi-quantitative culture method could be utilized to exclude the possibility of VAP, whereas the detection of abundant (≥3+) bacteria in semi-quantitative Gram stain could be utilized to suspect VAP. This study suggests merit for conducting further studies to evaluate the efficacy of semi-quantitative Gram stain and culture scoring results on antibiotic prescription behavior of physicians or clinical outcome of patients with clinically suspected VAP.

## Authors’ information

SH is an Assistant Professor in Anesthesiology and Intensive Care of Kyoto Prefectural University of Medicine, Japan. NS is a Visiting Associate Professor of Kyoto Prefectural University of Medicine, and Director of Trauma and Critical Care Center in National Hospital Organization Kyoto Medical Center.
